# Black and Red Currant Pomaces as Raw Materials to Create Smoothies with In Vitro Health-Promoting Potential

**DOI:** 10.3390/foods13172715

**Published:** 2024-08-27

**Authors:** Martyna Szydłowska, Aneta Wojdyło, Paulina Nowicka

**Affiliations:** Department of Fruit, Vegetables and Plant Nutraceutical Technology, Faculty of Biotechnology and Food Science, Wrocław University of Environmental and Life Sciences, 51-630 Wrocław, Poland; martyna.szydlowska@upwr.edu.pl (M.S.); aneta.wojdylo@upwr.edu.pl (A.W.)

**Keywords:** berry pomace, bioactive compounds, antioxidant activity, α-amylase inhibition effect, α-glucosidase inhibition effect, sensory evaluation

## Abstract

Pomace is a by-product resulting from the pressing of fruits and vegetables into juices, and it is typically treated as waste. Interestingly, pomace contains minimal amounts of protein and fat but is characterized by its high polyphenol and dietary fiber contents, which may have health benefits for human physiology. Therefore, they are a potentially attractive raw material for the food industry, but to our knowledge, no smoothies with their addition have been prepared and described so far. Consequently, products derived from apple juice, incorporating different doses of fresh (6% and 12%) and dried (3% and 6%) black or red currant pomace, were formulated, and their physical properties, chemical composition, bioactive compound content, and health-promoting potential (in vitro antioxidant and antidiabetic activity) were evaluated. Additionally, the products underwent sensory assessment by consumers. The fortified beverages exhibited different physical characteristics and chemical compositions than apple juice. All smoothies were characterized by higher concentrations of anthocyanins, flavonols, and procyanidin polymers compared to the base product. Moreover, 75% of them exhibited a significantly elevated phenolic acid content as well as a higher concentration of flavan-3-ols. The majority of fresh smoothies exhibited significantly higher in vitro antioxidant capacities and increased in vitro α-amylase and α-glucosidase inhibitory effects compared to the base product. The highest ABTS activity was recorded in the variant with 6% dried black currant pomace. In turn, the smoothie with 3% dried red currant pomace had the most effective FRAP effect and, together with the product containing 12% fresh black currant pomace, ORAC antioxidant activity and α-glucosidase inhibition also. The introduction of 6% dried red currant pomace led to the creation of a beverage that most effectively inhibited α-glucosidase. The study showed that the application of various types of pomace, mainly that of black currant, into apple juice enables the development of new functional products with sensory attributes that are favorably evaluated by consumers.

## 1. Introduction

The fruit and vegetable industry generates a significant amount of waste, necessitating the exploration of novel waste management approaches. Fruit pomaces represent the largest by-product of this sector, comprising fruit skins, seeds, and portions of pulp resulting from juice pressing or the production of wines and syrups. They typically account for 20 to 25% of the initial raw material weight. Interestingly, research suggests that they could serve as an appealing raw material for the production of new foods with health-promoting properties [1,2,3].

Pomaces contain a high carbohydrate content alongside organic acids, polyphenolic compounds, and vitamins while typically containing lower levels of protein and fat. As a result, they find utility across various industries, including in the extraction of dyes, phenolic compounds, pectin, and condensed aromas, as well as in animal feed production [2,4]. In addition, their phenolic compound content tends to surpass that of the whole fruit due to a significant portion being bound to the insoluble polysaccharides of the fruit’s cell walls, thus remaining in the waste during pressing [5]. For example, Kapci et al. [6] reported that chokeberry pomaces exhibited the highest levels of total phenolic compounds and anthocyanins compared to fresh and dried chokeberry, its juices, concentrates, jams, compotes, and syrups, as well as raspberry–chokeberry and cherry–chokeberry syrups. Therefore, pomace may be an attractive raw material for the food industry. Research has already been conducted on incorporating it directly into food recipes, such as biscuits and fruit teas [4], ciders [7], or breads [8].

A particularly intriguing avenue appears to be the use of berry fruit pomace in food production, such as that of black currants (*Ribes nigrum* L.) and red currants (*Ribes rubrum* L.). These fruits enjoy widespread popularity, with many consumers appreciating their flavors. In addition, the constituent compounds found in these fruits offer significant benefits to overall bodily function [9]. Research shows, that berry fruits may have a beneficial effect on postprandial blood glucose levels in humans. After consuming a meal consisting of 35 g of sucrose, 120 mL of tap water, and 150 g of mixed fruit purée (including bilberries, black currants, cranberries, and strawberries), a group of 12 people exhibited lower capillary and venous plasma glucose and serum insulin concentrations 15 min after their consumption, with higher concentrations observed after 90 min, compared to those who consumed a control meal with equivalent sucrose and carbohydrate amounts in a water solution [10]. Currants possess antioxidant and anti-rheumatic effects, as well as diuretic, digestive, and appetizing properties [11]. Furthermore, in vitro studies show that black and red currants have an inhibitory cyclooxygenase activity, which may indicate anti-inflammatory effects. The same work shows the ability of these fruits to inhibit acetylcholinesterase (AChE) and butyrylcholinesterase (BChE) [12]. Their inhibitions of AChE and BChE suggest that they may potentially prevent the development of neurodegenerative diseases such as Alzheimer’s and Parkinson’s diseases [13]. Additionally, the authors report that substances contained in black currant fruit may prevent the development of certain eye diseases and circulatory system diseases [14]. This suggests that products containing their pomaces could offer promising benefits as well.

Black currants are characterized by their particularly high content of vitamin C (114–264 mg/100 g of fresh raw material). They also contain sugars (5–8%), minerals (0.60–0.96%), dietary fiber (5%), and pectin (1%). Additionally, they are rich in polyphenolic compounds such as anthocyanins, phenolic acids, flavan-3-ols, and flavonols. Their organic acids include citric, malic, maleic, tartaric, amber, and salicylic acids. Additionally, they have a high water content (77–88%), contributing to their lower energy value [15,16,17].

Black currant fruit is associated with numerous health-promoting properties, including diaphoretic and antiscorbutic effects, immune system reinforcement, metabolism acceleration, and potential role in treatment for angina or gastric and duodenal ulcers [18,19,20].

On the other hand, red currants are a source of trans-resveratrol, dietary fiber (8%), phenolic compounds, and vitamin C. Unfortunately, their vitamin C content is estimated to be about four times lower compared to black currants. In addition, these fruits contain less potassium, calcium, phosphorus, magnesium, iron, retinol equivalent, β-carotene, vitamin E, and folate than black currant. Their energy value is also lower [19,20,21].

However, despite these differences, red currants may contribute to reducing the risk of osteoporosis, diabetes, cancer, hypertension, and other cardiovascular diseases [22]. In vitro studies showed that the red currants’ inhibition of cyclooxygenase-1 (COX-1), cyclooxygenase-2 (COX-2), AChE, and BChE was less effective compared to the black currants by 2%, 9%, 5%, and 16%, respectively. However, compared to other berries such as bilberries and blueberries, the COX-1 inhibitory effect of red currants was greater by 69 and 70%, respectively. In addition, the inhibition of AChE by red currants was 153% higher than by bilberries. Moreover, the inhibition of BChE by red currants was more effective than that by bilberries and blueberries by 44% and 210%, respectively [12].

Therefore, the aim of the present research was to create functional fruit beverages with health-promoting potential (in vitro antioxidant and antidiabetic properties) by enriching apple juice with fresh or dried black or red currant pomace. These beverages aimed to exhibit an increased polyphenolic compound content compared to the base product while remaining appealing to consumers. To date, no research has explored the feasibility of developing similar functional beverages through this approach.

This article presents the study of the physicochemical properties, bioactive compound content, antioxidant and antidiabetic properties, and sensory evaluations conducted on the smoothies obtained, with comparisons drawn between the parameters of the base juice.

## 2. Materials and Methods

### 2.1. Chemicals and Reagents

The enzyme preparation Pectinex YieldMASH was procured from Novozymes. NaOH was supplied by P.P.H. STANLAB (Lublin, Poland), while acetone, oxalic acid, and formic acid were sourced from CHEMPUR (Piekary Śląskie, Poland). Acetic acid, 6-hydroxy-2,5,7,8-tetramethylchroman-2-carboxylic acid (Trolox), 2,2′-azinobis-(3-ethylbenzthiazoline-6-sulfonic acid) (ABTS), 2,2′-azobis (2-amidino-propane) dihydrochloride (AAPH), fluorescein disodium (FL), potassium persulfate, TPTZ (2,4,6-tripyridyl-1,3,5-triazine), FeCl_3_, phloroglucinol, 3,5-dinitrosalicylic acid, potassium sodium tartrate tetrahydrate, sodium phosphate monobasic, potato starch, α-amylase, dipotassium hydrogen orthophosphate dihydrogen, p-nitrophenyl-α-D-glucopyranoside, α-glucosidase, standards of sugars, and methanol were purchased Sigma-Aldrich (Steinheim, Germany). Standards of organic acids and polyphenols were sourced from Extrasynthese (Lyon Nord, France). Chlorogenic acid was provided by TRANS MIT GmbH (Giessen, Germany). Acetonitrile for ultra-pressure liquid chromatography (UPLC, gradient grade) was purchased from Merck (Darmstadt, Germany). UPLC-grade water, prepared using the HLP SMART 1000s system (Hydrolab, Gdańsk, Poland), was further purified through a 0.20 µm membrane filter (Millex Samplicity^®^ Filters Membrane) immediately before use.

### 2.2. Sample Preparation

Black currants (*Ribes nigrum* L.) and red currants (*Ribes rubrum* L.) were collected from the plantation in Próba, located near Sieradz (Poland) (51.5123862 N, 18.6494130 E). The bushes were irrigated and subjected to standard farming practices (pruning, thinning, fertilization, and pest control treatments). Undamaged berry fruits were hand-harvested at optimum ripeness at the end of June 2023 (cultivars ‘Tisel’ (black currant) and ‘Detvan’ (red currant)). Immediately after harvesting, whole fruits were processed.

Berry fruits underwent destalking, washing, homogenization, and enzymatic treatment (t = 30 min; T = 23 °C) using the enzyme preparation Pectinex YieldMASH at a concentration of 0.05% (with a ratio of enzyme preparation-to-tap water of 1:9). Subsequently, the fruits were pressed to yield pomace and raw juice.

Fresh pomace was incorporated into the apple juice at levels of 6% or 12% and thoroughly mixed. The mixture underwent homogenization using a Vorwerk Thermomix (t = 1 min), followed by pasteurization (T = 98 °C) and another round of homogenization (t = 1 min). The resulting products were then hot filled into glass jars, subjected to self-pasteurization (t = 10 min), and cooled in a cold laboratory water bath (t = 10 min).

The preparation process for products containing dried pomace followed a similar procedure, except that the pomace was dried using an air dryer (t = 240 min; T = 75 °C), then ground in a Bosch electric grinder, and finally, mixed with the juice. Dried pomace was added in amounts of 3% or 6%.

To determine the pomace-to-juice ratio for developing smoothies with acceptable sensory characteristics, several variants with different doses of fresh pomace were prepared and evaluated. The researchers decided to use the doses that were rated well in both cases. Products with dried additives were created as their equivalents. The mass of pomace decreased by approximately 50% during drying; hence, their quantities in the products were comparable. Their addition can be categorized as a single dose (3% dried raw material or 6% fresh) or a double dose (6% dried or 12% fresh).

In total, eight variants of smoothies were prepared: with the addition of a single or double dose of dried and a single or double dose of fresh black currant pomace, as well as with the addition of a single or double dose of dried and a single or double dose of fresh red currant pomace. Apple juice served as the control.

### 2.3. Physical Parameters

#### 2.3.1. CIEL*a*b* Color Measurement

Color measurements were conducted using the ColorQuest XE (HunterLab, Reston, VA, USA), following the guidelines provided in its user manual. Measurements were performed in transmitted light with 1 cm thick cuvettes for a 10° observer and a D65 illuminant. Each sample was measured twice, and the results are presented as the average value ± SD.

#### 2.3.2. Dynamic Viscosity

The dynamic viscosity was measured using a Brookfield DV II Pro viscometer (Middleborough, MA, USA) equipped with an S 63 spindle (t = 0.5 min; RPM = 100). Each measurement was repeated twice, and the results are presented as the average value ± SD.

#### 2.3.3. Turbidity Stability

Turbidity measurements were carried out using a HANNA Instruments device (HI 93703 Microprocessor Turbidity Meter, Woonsocket, RI, USA). The sediment content in the products before (B) and after (A) centrifugation in the MPW 250 laboratory centrifuge was measured (t = 5 min; T = 6 °C; RPM = 10,000). The results are expressed as (A/B) × 100 [% NTU].

### 2.4. Chemical Ingredients

#### 2.4.1. Basic Chemical Composition

Dry matter analysis was conducted following the PN-EN 12145:2001 standard [23]. Extract analysis was conducted using a refractometer with a measurement range of 0.53°Brix (ATAGO Pocket PAL-1, Bellevue, WA, USA) and in accordance with the PN-EN 12143:2000 standard [24]. The total acidity was measured via potentiometric titration following the PN-EN 12147:2000 standard [25]. The pH value and the amount of NaOH used were recorded using the TitroLine 6000 titrator (SI Analytics, Mainz, Germany). Total acidity was expressed as the content of malic acid [g]/100 g of the product. Ash content analysis was performed according to the PN-EN 1135:1999 standard [26]. The pectin content was determined following the procedure described by Pijanowski et al. [27]. All measurements were performed with replicates and are presented as the average value ± SD.

#### 2.4.2. Sugar Content Using HPLC-ELSD Method

Approximately 6 g of the test product was weighed into a volumetric flask. The sample was then quantitatively transferred using distilled water, with the volumetric flask filled to one-third of its volume, and subsequently placed in a GFL 1083 laboratory water bath (Dahej, India). The water in the device was heated to a temperature of 98 °C and maintained for 20 min. Afterward, the volumetric flask was cooled in a cold laboratory water bath, topped up to the mark with distilled water, and thoroughly mixed. The sample was then transferred to a centrifuge tube and centrifuged in a MPW 250 laboratory centrifuge (Warszawa, Poland) (t = 5 min; T = 6 °C; RPM = 10,000). The supernatant was decanted using filter papers and purified on sepaks placed in a BAKER SPE 12G vacuum system (J.T. Baker, Phillipsburg, NJ, USA) with a KNF NEUBERGER LABOPORT vacuum pump (KNF Neuberger GmbH, Freiburg im Breisgau, Germany). Subsequently, the sample underwent vacuum filtration using filters with a diameter of 0.02 mm (Millex Samplicity Filter, Merck, Darmstadt, Germany).

Chromatographic analysis was conducted following the method outlined by Wojdyło et al. [28]. The results of the sugar content (fructose, sorbitol, glucose, and sucrose) are presented as the average value (g/100 g of product) ± SD.

#### 2.4.3. Organic Acid Content

Approximately 6 g of the tested product was quantitatively mixed with distilled water in a volumetric flask, bringing the volume to one-third of the flask’s capacity. The mixture was then heated in a GFL 1083 laboratory water bath (t = 20 min; T = 98 °C), followed by cooling in a cold laboratory water bath. After topping up the flask to the mark with distilled water and thorough mixing, the sample underwent centrifugation in a MPW 250 laboratory centrifuge (t = 5 min, T = 6 °C; RPM = 10,000). The supernatant was decanted using filter papers. The samples were initially purified using a BAKER SPE 12G vacuum system with a KNF NEUBERGER LABOPORT vacuum pump and then using 0.02 mm diameter filters (Millex Samplicity Filter, Merck). Chromatographic analysis was conducted following the method described previously by Wojdyło et al. [29]. The result was expressed as the average content of organic acid [g] per 100 g of product ± SD.

#### 2.4.4. Content of Polyphenolic Compounds, Including Polymers Procyanidins, Using UPLC

The product samples were centrifuged using an MPW 250 laboratory centrifuge (t = 5 min; T = 6 °C; RPM = 10,000). The resulting supernatants were then purified under vacuum using 0.02 mm diameter filters. Chromatographic analysis was conducted following the method outlined by Nowicka et al. [30]. The content of anthocyanins, phenolic acids, flavonols, and flavan-3-ols was quantified in mg/100 mL of sample using coefficients prepared for selected standards of the discussed compounds. The results are presented as the average value ± SD.

Polymeric procyanidins analysis was performed using the phloroglucinol method following the protocol described by Kennedy and Jones [31]. About 0.500 g of the samples were weighed into the tubes (Eppendorf, Hamburg, Germany), covered with parafilm, punctured, frozen, and dried via sublimation using a Labconco FreeZone TRAY DRYER freeze dryer (Kansas City, MO, USA) with an EDWARDS RV8 vacuum pump. Next, the Acquity UPLC System liquid chromatograph (Waters Co.; Milford, CT, USA) and a fluorescence detector were used for the analysis. The data were compiled using Empower^®^ 3 software. Calibration curves were prepared using epicatechin, (+)-catechin, and procyanidin B1. The results are presented as the average value (mg/100 mL) ± SD.

### 2.5. Analysis of Health-Promoting Potential Using In Vitro Methods

#### 2.5.1. Antioxidant Activity

The ABTS•+ antioxidant activity assay, as described by Re et al. [32], relies on the reduction of the blue-green color of ABTS•+ by antioxidant substances. The decrease in color intensity correlates directly with the concentration of antioxidants in the solution. Absorbance measurements were conducted after 6 min at a wavelength of *λ* = 734 nm using a UV 2401 PC spectrophotometer (Shimadzu, Kyoto, Japan).

The Ferric Reducing Ability of Plasma (FRAP) assay, outlined by Benzie and Strain [33], is based on the ability of antioxidant substances to reduce Fe^3+^ complexed with tripyridyl triazine (TPTZ) to Fe^2+^, resulting in a color change from colorless to blue. Absorbance was measured after 10 min at a wavelength of *λ* = 593 nm using a UV 2401 PC spectrophotometer (Shimadzu, Japan).

The Oxygen Radical Absorbance Capacity (ORAC) assay, described by Ou et al. [34], involves the decay of fluorescence of a fluorescent substance (typically fluorescein) due to oxidation by free radicals. Antioxidants prevent changes in fluorescence, thereby inhibiting the oxidation of the fluorescent substance. The antioxidant activity was measured using an RF5301 PC spectrofluorometer (Shimadzu, Kyoto, Japan) at an excitation wavelength of 487 nm and an emission wavelength of 528 nm. Plates were incubated at 37 °C, with measurements taken every 5 min following the introduction of the AAPH reagent. The duration of analysis depended on the rate of fluorescence decay.

The results for all methods are expressed as mmol of Trolox/100 mL of sample ± SD, based on three repetitions.

#### 2.5.2. Ability to Inhibit α-Amylase and α-Glucosidase

The analyses were performed as described previously by Nowicka et al. [30].

The method employs a color reaction, where iodine causes amylopectin to turn purple and amylose to turn blue. Both structures are highly polymerized. The introduction of the α-amylase enzyme to the sample containing starch leads to depolymerization and the subsequent loss of color. The ability to inhibit α-amylase activity was determined by measuring the absorbance using a UV 2401 PC spectrophotometer (Shimadzu, Kyoto, Japan) at a wavelength of 540 nm. The second method involves measuring the amount of glucose hydrolyzed by p-nitrophenyl-α-D-glucopyranoside. Spectrophotometric measurements for assessing the ability to inhibit α-glucosidase were performed using the same spectrophotometer at a wavelength of 405 nm. In both analyses, acarbose was used as a positive control. The obtained results are shown as IC_50_ (mg/mL ± SD).

### 2.6. Sensory Evaluation

The sensory evaluation took place at the Faculty of Biotechnology and Food Sciences of Wrocław University of Environmental and Life Sciences, following the ISO 8589:2009 standards [35]. The panelists consisted of students specializing in Food Technology and Human Nutrition. They underwent comprehensive training, and as per national regulations, ethical approval was not necessary for this study. The panelists volunteered to participate and were briefed on the study’s objectives, with the option to withdraw from the evaluation at any time.

Analyses of the color, aroma, and overall impression of the products were carried out using a 5-point hedonic scale, where 1 indicated dislike very much and 5 indicated like very much. Additionally, the consistency of the products was evaluated on a scale where 1 represented very liquid and 5 represented very thick. The number of discernible fruit flavors was determined through a multiple-choice questionnaire regarding five items: apple, black currant, chokeberry, raspberry, and red currant.

The study was based on the guidelines of the Declaration of Helsinki 18. The personal data of the participants of the organoleptic evaluation were coded following the guidelines of the General Regulation of the European Parliament on the Protection of Personal Data (GDPR 679/2016). No ethical approval was required for this study because of national laws (Journal of Laws 1999, No. 47, item 480, and Journal of Laws 1997, No. 28, item 152). Participants were informed that their participation was entirely voluntary so that they could stop the analysis at any point and that the responses would be anonymous.

### 2.7. Statistical Analysis

Statistical analysis was conducted using Statistica software version 13.3. To compare the mean parameter values among the products, a three-factor or four-factor analysis of variance (ANOVA) was performed, followed by the Duncan test (*p* ≤ 0.05).

The relationships between the selected product components and physicochemical properties or in vitro health-promoting potential were determined using the Pearson correlation coefficient (*r*).

## 3. Results and Discussion

### 3.1. Basic Physical Properties of the Obtained Smoothies

Consumer perception of food is heavily influenced by its color. Particularly with products displayed in transparent packaging, an unfavorable assessment of color can lead to hesitancy in purchase decisions. Consumers understand that a food’s color reflects its quality and composition. The darkening of juices, for example, may indicate enzymatic browning, which degrades phenolic compounds and reduces their health-promoting properties. Additionally, products may undergo the Maillard reaction or sugar caramelization [36]. Moreover, the intensity of color is influenced by the compound concentration, qualitative composition, and pH, which impacts stability [6,37].

The colors of the prepared smoothies were measured in the CIEL*a*b system (Table 1). The analysis revealed no significant differences among the individual products. The darkest variant was the one containing 12% fresh black currant pomace (34.80), while the lightest was the product with 12% fresh red currant pomace (50.86). Smoothies with red currant pomace appeared lighter than pure apple juice (37.48), whereas most products with black currant pomace were darker. One study showed that the flesh of black currants is lighter than their outer parts [38], suggesting that the incorporation of their pomace into juice may lead to a more pronounced reduction in the brightness of the final products compared to using the whole fruit. Additionally, the use of dried pomace resulted in significantly darker products than with fresh pomace. The L* parameter increased significantly in products with single and double doses of pomace, with no significant difference based on the dose size. However, the color of the smoothies notably lightened after storage.

The a* and b* parameter values obtained for each product were positive, indicating that all colors tended towards red and yellow. All smoothies exhibited a more pronounced red hue compared to the base juice, hinting at a potential increase in their anthocyanin content. Research has shown that the anthocyanin content in chokeberry pomace, another type of berry fruit, was twice as high as in fresh raw material [6]. Consequently, the observed intensification of red color in these smoothies may exceed what would be expected with the addition of fresh berries alone.

The product containing 6% dried red currant pomace (16.60) exhibited the most intense red color, whereas the smallest increase in intensity compared to the base juice (0.32) was observed with the addition of 6% fresh red currant pomace (12.50). However, even this increase, which was the smallest, had statistical significance.

Smoothies with black currant pomace displayed notably more intense red coloring. Each incremental dose of the additive corresponded to a statistically significant increase in the color intensity. Additionally, storage resulted in a significant decrease in the value of the a* parameter in the products. However, given the instability of anthocyanins, the observed color changes, influenced both by the type of pomace used and by storage, primarily reflect the presence of these compounds [39].

The dynamic viscosity of the products was also examined, and the results are presented in Table 1. In liquid products, viscosity is influenced by both soluble and insoluble fractions of dietary fiber and sugars. Pasteurization further impacts viscosity, as it reduces the pectin and fiber contents compared to freshly pressed products [40]. The measurements showed that the differences in dynamic viscosities among the products were not statistically significant. Storage did not significantly affect the dynamic viscosity either. However, products containing black currant pomace exhibited significantly higher values compared to those with red currant pomace, suggesting a higher content of the aforementioned ingredients. The dynamic viscosity was significantly higher in products with fresh pomaces compared to dried ones. Each incremental addition resulted in smoothies with significantly higher viscosities. Additionally, all values exceeded those reported by Mot et al. [40] for clarified juices from beetroot, apple, carrot, celery, and orange (1.2, 1.4, 1.4, 1.5, and 1.5 mPas, respectively), as well as their mixture (1.3 mPas). Similar values were achieved by Sitkiewicz et al. [41] for clarified apple and chokeberry juices, which were 1.4 mPas in both cases. However, the dynamic viscosities of the prepared smoothies did not surpass those of non-macerated apple purée reported in other studies, i.e., 2900.0 mPa [42].

The stability of the turbidity in the created products was analyzed (Table 1). Particles suspended in higher-quality cloudy juices tend to remain suspended longer. Their size and the fluid viscosity are influenced by the ripeness of the raw material used. Juices are deemed stable if the ratio of their turbidity after centrifugation to the turbidity of the uncentrifuged sample is above 50% and the stable turbidity level is at least 250–300 NTU [36]. The most stable, compared to others, was the stored smoothie with a double dose of dried black currant pomace (22.82% NTU). Next were the unstored apple juice and variants with 6% dried and fresh black currant pomace (19.04, 18.95, and 18.42% NTU, respectively), with no statistically significant differences between them. These results were lower than those obtained by other authors for apple juice from the Rajka variety enriched with vitamin C (49.82% NTU) and more consistent with the values measured for the variant from the same variety but without the addition of ascorbic acid (7.02% NTU) and for juice from the Jonathan variety with vitamin C (10.68% NTU) [36]. Additionally, the type of pomace significantly impacted this parameter in the tested products. Smoothies with black currant pressing waste exhibited greater stability. The use of dried raw materials also had a significantly more beneficial effect on shaping this parameter. Fortifying the juice with pomace, whether with a single or double dose, resulted in smoothies with significantly lower average stabilities, with no significant differences between them. Additionally, storage significantly reduced the turbidity stability of the products.

The measurements showed that the turbidity stability observed in the prepared variants did not surpass the critical value of 50%, confirming a high probability of sedimentation of suspended particles.

### 3.2. Basic Chemical Properties

The dry matter content of a product is indicative of its quality, nutritional value, and suitability for storage [43]. In all tested product variants with pomace additions, the dry matter content was higher (14.22–18.72%) compared to the base juice (unstored: 13.28%; after storage: 13.03%) (Table 1). This could be attributed to pomace being a raw material characterized by a high concentration of solid ingredients. For example, Banaś and Korus [44] reported that apples contain 15.10% dry matter. Research by Wojdyło et al. [45] indicated that depending on the apple cultivation type, these fruits contain 17.46–17.76% dry matter. In contrast, Wichrowska and Żary-Sikorska [46] reported the dry matter content in apple pomace to be 31.80%, and in freeze-dried apple pomace, it is 92.90%. Additionally, fruit selection may also have had a significant impact. Other authors reported, for example, that apple mousse contained a smaller amount of dry matter (18.32%) compared to apple–black currant mousse (21.38%) [47].

Statistical analysis also showed that smoothies with red currant pomace had a significantly higher dry matter content compared to those with black currant pomace residue. Moreover, a notably higher concentration of dry matter was observed when using the dried additive. With each increment in pomace quantity, the beverages showed a significantly elevated dry matter content. However, after storage, the dry matter content decreased in the products.

Total extract measurement allows for determining the number of substances soluble in water and nonvolatile with water vapor, including inorganic substances, nitrogenous organic substances, and nonvolatile organic acids. These components contribute to the sensory properties and nutritional quality of the food [48]. The results obtained are presented in Table 1.

Among the unstored products, the smoothie with 12% fresh black currant pomace had the lowest total extract (12.1°Brix) (Table 1). However, the values measured in the other variants (12.5–14.8°Brix) were higher than those in the base juice (12.3°Brix).

Generally, products with red currant pomace had a significantly higher total extract value compared to those with black currant pressing waste. Discrepancies in total extract values are evident in studies by other authors depending on the raw material used. For example, Szajdek et al. [47] showed that the extract of apple–black currant mousse (20.5%) exceeded that of apple mousse (18.5%).

Additionally, in the present study, a significantly higher total extract value was observed after using dried pomace. With each increment in the additive dose, the products exhibited a significantly higher total extract value. Interestingly, after storage, the total extract content in the tested beverages increased.

The total acidity, pH, ash, and pectin contents of the products were also analyzed. The results of these analyses are summarized in Table 1.

A higher total acidity in food may signify a greater content of organic acids in the raw materials used for production. Conversely, an increase in the total acidity during storage could indicate fermentation, while its decrease, as observed in wines, might result from the precipitation of dye–tannin compounds [19]. The concentration of hydrogen ions determines the pH [43]. Importantly, anthocyanins, for example, remain stable at pH levels of 3.5 and below but degrade at more alkaline pH levels than those mentioned above [39].

Statistical analysis showed that the differences between the results obtained for individual products before and after storage regarding total acidity (0.35–0.74 g malic acid/100 g) were not statistically significant. However, in the case of the pH, differences were observed. The lowest pH value was significantly recorded in the unstored product with a 6% addition of dried black currant pomace (pH: 3.060). Conversely, the significantly highest pH values were determined in stored smoothies with the addition of 12% fresh red currant pomace (pH: 3.427), 6% fresh red currant pressing waste (pH: 3.407), and 3% dried black currant pomace (pH: 3.406), as well as in the stored apple juice (pH: 3.409), with no statistically significant differences among them.

The choice of pomace, its processing, dose, and beverage storage significantly influenced the total acidity and pH. Smoothies with black currant pomace had a higher total acidity and lower pH (more acidic reaction) compared to those with red currant pomace. Other authors reported that in the case of fruit purées, apple sauce had a total acidity of 0.43 g/100 g, while apple–currant purée containing black currant had a total acidity of 1.67 g/100 g [47]. These data underscore the significance of raw material selection in shaping the acidity of the final product.

Moreover, the use of dried additives in this study resulted in products with higher acidity, as confirmed by both methods. Each increase in the dose had a significant impact on acidity enhancement in the beverages. Both analytical approaches also showed that storage significantly decreased the acidity of the tested beverages.

The study also assessed the mineral content, represented as ash. Its presence in food indicates its nutritional value [43]. However, statistical analysis indicated no statistically significant differences between the individual values obtained. The ash contents in unstored and stored products ranged from 0.18 g/100 g to 0.31 g/100 g and 0.17 g/100 g to 0.35 g/100 g, respectively. However, it was observed that each increase in the pomace dose resulted in the production of beverages with significantly higher ash contents.

The products were subjected to testing for their pectin content, which, akin to marine plant polysaccharides and plant gums and glues, represents a soluble fraction of dietary fiber [49]. Pectin holds considerable significance in human nutrition, as it contributes to the prevention of obesity, diabetes, and cardiovascular diseases [50]. Notably, pectin is characterized by a high viscosity, enhancing the feeling of satiety upon consumption of foods containing them. Moreover, this product remains in the stomach for an extended period, facilitating prolonged nutrient absorption [51].

Statistical analysis of the prepared products with pomace revealed no statistically significant differences in the individual pectin contents of the beverages (0.00–0.10%). Nevertheless, each increase in the pomace addition size resulted in beverages containing significantly higher amounts of pectin.

### 3.3. Sugar and Organic Acid Contents Using HPLC

The tested smoothies exhibited varying sugar contents (Table 2). Among them, the smoothie with a 12% addition of fresh black currant pomace contained the lowest total sugars, with the lowest concentrations of fructose and sorbitol. Additionally, along with the variant containing 6% fresh black currant pomace and apple juice, it fell into the category of products with the least glucose, and, along with the smoothies containing 6% and 12% fresh red currant pomace, it was one of the variants without sucrose.

The variants containing 3% and 6% dried red currant pomace, showed statistically significantly higher total sugar contents (16.33 and 17.26 g/100 g, respectively) compared to apple juice (14.33 g/100 g). Conversely, the remaining variants exhibited statistically significantly lower or similar contents (12.44–14.76 g/100 g). Moreover, these two variants and the smoothie with 6% fresh red currant pomace also had a higher fructose content (13.28, 13.97, and 12.31 g/100 g, respectively) compared to the base juice (11.85 g/100 g). Interestingly, research by Jurgiel-Małecka and Buchwał [16] indicated an inverse relationship, with black currant fruits (42.72 g/100 g dry matter) containing a higher total sugar content compared to red currant fruits (42.03 g/100 g dry matter). However, discrepancies between the compositions of the fruits could stem from differences in their varieties, harvesting periods and years, places of cultivation, or general growing conditions [9,11,52].

In this study, the utilization of all pomaces led to the creation of products with similar or higher glucose contents (2.12–2.97 g/100 g) compared to apple juice (2.18 g/100 g). Conversely, only the variant incorporating 3% dried red currant pomace (0.29 g/100 g) exhibited a significantly higher sucrose content than the base juice (0.22 g/100 g).

The smoothies containing pomace had a higher sugar content compared to the products examined by Lebiedzińska et al. Their study showed that the apple juice they freshly pressed contained an average of 10.60 g of sugar/100 g, while the purchased one ranged from 8.68 to 10.16 g/100 g. Furthermore, the juices they extracted from tangerines, grapefruits, oranges, lemons, limes, kiwis, and pears also exhibited lower sugar contents than the products evaluated in this research [53].

Statistical analysis showed that the use of black currant pomace led to smoothies with significantly lower contents of fructose, sorbitol, glucose, and total sugars compared to the use of red currant pomace. Prior drying of the pressing waste also had a notable effect, resulting in products with higher concentrations of fructose, sorbitol, glucose, sucrose, and total sugars.

Additionally, the relationship (*r*) between the sugar content and dynamic viscosity was examined and presented in Supplementary Table S1. The analysis indicated moderate relationships between viscosity and the amounts of fructose (*r* = −0.64), sorbitol (*r* = −0.46), sucrose (*r* = −0.68), and total sugars (*r* = −0.63).

The authors report that the organic acid content in apple juices may vary depending on factors such as the variety, origin of the raw material, climate, fruit storage method, processing techniques, and juice storage conditions [54]. These same factors could influence the acid profiles of other food products, including the tested smoothies.

The base juice examined in this study contained citric acid (0.05 g/100 g) and malic acid (0.96 g/100 g) (Table 2). Utilizing pomace resulted in products with higher concentrations of these acids, and depending on the variant, also oxalic acid, maleic acid, quinic acid, and/or shikimic acid.

The use of black currant pomace generated products with significantly elevated levels of oxalic, malic, and quinic acids and lower levels of maleic, citric, and shikimic acids compared to those with red currant pomace. The introduction of fresh pomace led to a smoothie with significantly higher concentrations of oxalic, citric, and malic acids. However, drying did not have a statistically significant effect on the content of maleic, quinic, and shikimic acids in the products. Each increment in the pomace addition size resulted in a significant rise in the amounts of maleic, citric, malic, and shikimic acids in the beverages. While the differences in the oxalic and quinic acid concentrations between the base juice and products enriched with single doses were not statistically significant, their quantities in smoothies with a double dose of pressing waste were significantly higher.

It was found that the relationship (*r*) between the total organic acid content and pH value, as well as between the former and total acidity (Supplementary Table S2), is moderate (*r* = −0.53 and *r* = 0.56, respectively). Among individual acids, the strongest correlation (the largest compared to the others) was observed between the amount of citric acid and total acidity, which amounted to *r* = 0.63.

The total organic acid content in the smoothies immediately after preparation ranged from 2.19 to 4.65 g/100 g, and after three months of storage, it decreased to a range of 1.11 to 1.95 g/100 g. The content was even lower in the unenriched juice, with 1.01 g/100 g in the case of the unstored juice and 0.87 g/100 g in the juice after storage. According to statistical analysis, the effect of storage on reducing the total organic acid content was statistically significant.

A decrease in the organic acid content was also observed by other authors conducting research on concentrated apple juices. They suggested that the likely cause could be combinations of organic acids with compounds resulting from browning [54].

### 3.4. Bioactive Compound Contents in the Obtained Smoothies

Polyphenolic compounds affect the taste and color of products, reduce the activity of pectinolytic enzymes, and cause clouding of liquid products [3,42,55]. They are renowned for their antioxidant, anticancer, antidiabetic, and various other properties [6,20,22,50,56].

Products with pomace addition exhibited significantly higher polyphenolic compound contents than the base juice (Table 3). The total polyphenol content was significantly higher in variants with black currant pomace than those with red currant pomace. The literature also indicates that black currant fruits are richer in these compounds than red currant fruits. Gryszczyńska et al. [57] reported their content in the fruits, depending on the variety, as 535.50–888.50 mg/100 g and 371.00–501.60 mg/100 g, respectively. Conversely, research by Jurgiel-Małecka and Buchwał [16] showed their content in black currants as 1709.00 mg/100 g of dry matter, in red currants as 587.00 mg/100 g of dry matter, and in white currants as 401.00 mg/100 g of dry matter. Additionally, in this study, products with the dried addition were characterized by significantly higher total polyphenolic compound contents than smoothies with fresh pomace.

The type of pomace used had a significant impact on the content of anthocyanins, phenolic acids, flavonols, flavan-3-ols, and procyanidin polymers. Smoothies with the black currant pomace addition exhibited notably higher anthocyanin, flavonol, and procyanidin polymer contents. Conversely, smoothies with red currant pomace contained higher amounts of phenolic acids and flavan-3-ols. Pomace drying also played a significant role in the composition of the beverages. Smoothies with fresh pressing waste had a higher anthocyanin concentration, whereas products with dried additives showed higher concentrations of phenolic acids, flavan-3-ols, and procyanidin polymers. Each increase in the pomace dose resulted in a statistically significant increase in the anthocyanin, flavonol, and procyanidin polymer contents and a decrease in the phenolic acid content. Additionally, storage significantly reduced the concentrations of anthocyanins, phenolic acids, flavonols, and procyanidin polymers in the tested products while leading to an increase in the flavan-3-ol content. This increase was likely due to their formation from procyanidin polymers.

The main group of polyphenolic compounds in the tested products was procyanidin polymers. Initially, the content of this fraction ranged from 39.24 mg/100 mL (in apple juice) to 135.92 mg/100 mL (in the variant with 12% fresh black currant pomace). Following storage, the base juice still exhibited the lowest concentration of procyanidin polymers (32.09 mg/100 mL), while the highest concentration was observed in the variant with 6% dried black currant pomace. Notably, an increase in the content of these compounds was noted (from 133.70 mg/100 mL to 161.70 mg/100 mL) in this case.

Additionally, the smoothies contained over 3 times (in the case of 3% dried red currant pomace) to almost 38 times more anthocyanins (in the case of 12% fresh black currant pomace) compared to the base juice (0.34 mg/100 mL). After storage, their content decreased, and the difference compared to the juice widened. Subsequently, the variant with 12% fresh black currant pomace contained an almost 120 times higher concentration of anthocyanins than the base juice.

Numerous factors influence the stability of anthocyanins, including the pH, light, temperature, oxygen enzymes, metal ions, ascorbic acids, proteins, sugars, and sulfur dioxide [39]. Therefore, the relationships between their concentration and the pH, individual sugars, and total sugars were examined for the tested products (Supplementary Table S3). It was revealed that the relationships between the anthocyanin concentration and pH (*r* = −0.72) or sorbitol (*r* = −0.72) were strong, while the relationships with fructose (*r* = −0.69), glucose (*r* = −0.51), and total sugars (*r* = −0.66) were moderate.

The impacts of the type and quantity of pomace and product storage on the anthocyanin content mirrored the results observed in the measurement of the a* parameter. However, the relationship between red color intensity and the anthocyanin concentration was moderate (*r* = 0.48), similar to the correlation between the a* value and flavonol concentration (*r* = 0.47), procyanidin polymers (*r* = 0.46), and total polyphenolic compounds (*r* = 0.48). These correlations are presented in Supplementary Table S4.

The data collected thus far suggest that due to the higher polyphenolic compound content in smoothies with black currant pomace or products with dried pressing waste, these variants may have greater health-promoting potential. Further investigation was undertaken to determine if the prepared beverages exhibited greater antioxidant and antidiabetic effects in vitro compared to the base juice.

### 3.5. Health-Promoting Potential

Consuming foods rich in antioxidants is important for maintaining the body’s proper functioning. These compounds play a vital role in neutralizing free oxygen radicals, which can form as a result of ionizing or ultraviolet radiation, exposure to chemicals, and other factors, posing potential harm to our health. When the body is in a state of homeostasis, free radicals either degrade or become involved in subsequent biochemical processes, minimizing their impact. However, if the redox balance is disrupted, they can cause damage to cellular and tissue structures, potentially leading to the development of cancerous changes [5,21,58]. It is important to note that the antioxidant capacity of products is influenced not only by the quantity of antioxidants present but also by the activity of enzymes that prevent oxidation [59].

Additionally, research conducted by Skąpska et al. [60] showed that apple juice subjected to centrifugation and pasteurization, fruit pulp, enzyme-treated pulp, raw juice, centrifuged juice, juice pasteurized after enzyme treatment, filtered juice, or microfiltered juice exhibited lower ABTS antioxidant activities (0.24–0.43 mg Trolox/1 g fresh weight) compared to the raw material (1.35 mg Trolox/1 g fresh weight). The most similar value to the raw material was observed in the concentrated juice (1.31 mg Trolox/1 g fresh weight). These findings suggest that antioxidant compounds may accumulate in the solid parts of the raw material.

To ensure the most accurate assessment of the antioxidant activities of smoothies, the research employed three distinct methods: ABTS, FRAP, and ORAC, each with its unique mechanisms. The findings showed that most products containing pomace exhibited superior antioxidant properties compared to the base juice, as indicated in Table 4.

The analysis of the ABTS antioxidant activity demonstrated that all smoothies, after preparation, showcased a significantly higher antioxidant efficacy than juice. Meanwhile, the FRAP study showed that all unstored products, except the variant with 3% dried black currant pomace, displayed greater antioxidant effects than apple juice. The difference between the mentioned variant and the juice was not statistically significant. The ORAC method highlighted that all smoothies, after preparation, exhibited a stronger antioxidant effect than the base juice, with statistically significant differences observed in all variants except for the one featuring 6% dried red currant pomace. Notably, the values obtained in this study surpass those reported in apple fruit by other authors using the ABTS method, which range from 0.005 to 0.006 mmol Trolox/100 g depending on the crop type [45].

The utilization of the ABTS and ORAC methods demonstrated a significantly greater increase in the antioxidant effects of the final products with the incorporation of black currant pomace compared to the addition of red currant pressing waste. Conversely, FRAP analysis showed the opposite trend. Furthermore, both the ABTS and FRAP assays indicated a significant increase in the antioxidant activities of the products with each increment in the pomace dose. With the ORAC method, it was observed that after the application of pomace, the resulting beverages exhibited heightened antioxidant properties compared to the base juice, with no significant differences in activity between the variants with single and double doses. Additionally, the drying process of the raw material had a significant impact on the results obtained using the ABTS and FRAP methods. Smoothies with dried additives displayed significantly higher antioxidant effects than beverages with fresh pomace. Across all three methods, there was a notable decline in the antioxidant properties of the products following storage.

After three months of storage, the differences in the activity levels between the smoothie and the base juice became statistically insignificant in many instances for individual products. This scenario occurred in three out of eight products with pomace addition during the ABTS analysis and in one out of eight during the FRAP analysis. Notably, the most significant attenuation of the antioxidant effect was observed in the ORAC method. The activities of five out of eight stored smoothies did not significantly differ from that of the stored base juice, whereas the activities of three out of eight products were notably lower than that recorded for the juice. This decline can be attributed to the decreased content of polyphenolic compounds in the products.

The Pearson correlation coefficient (*r*) revealed moderate relationships between the ABTS test results and the contents of anthocyanins (*r* = 0.58), procyanidin polymers (*r* = 0.58), flavonols (*r* = 0.44), and the total polyphenolic compound (*r* = 0.61). Regarding the FRAP method, moderate relationships were observed between its results and the concentrations of procyanidin polymers (*r* = 0.47), flavan-3-ols (*r* = 0.45), as well as total polyphenolic compounds (*r* = 0.49). Conversely, the results obtained using the ORAC test were moderately dependent on the amounts of anthocyanins (*r* = 0.60), flavonols (*r* = 0.46), procyanidin polymers (*r* = 0.41), and total polyphenols (*r* = 0.44). The relationships between the polyphenolic compounds content and the antioxidant activity of products are presented in Supplementary Table S5.

Additionally, the examined products underwent analysis for their antidiabetic properties, specifically their ability to inhibit α-amylase and α-glucosidase. These digestive enzymes, naturally occurring in humans in the pancreas and intestines, respectively, are responsible for breaking down carbohydrates such as oligosaccharides and disaccharides through hydrolysis. The resulting monosaccharides are then absorbed by the body, leading to a glycemic effect characterized by an increase in blood glucose levels. Inhibiting the hydrolysis of complex sugars can be particularly beneficial for individuals with diabetes, who, due to the impaired production or secretion of insulin, must carefully monitor their glycemic levels to maintain appropriate blood sugar levels. Insulin, a hormone crucial for carbohydrate metabolism, also influences fat and protein metabolisms, and acute hyperglycemia can pose serious health risks, potentially requiring hospitalization or leading to fatality. Therefore, it is especially important for them that postprandial glycemia increases gradually rather than suddenly. Prior research has highlighted the ability of flavan-3-ols to inhibit α-amylase and α-glucosidase. Research shows that extracts containing procyanidin polymers can reduce the α-amylase activity by forming complexes with enzyme molecules. Moreover, the antidiabetic effect of food may also be attributed to the presence of anthocyanins or flavonols [49,50].

The research results regarding the ability of the examined products to inhibit α-amylase and α-glucosidase were expressed as IC_50_, representing half of the maximum concentration that inhibits the action of a given substance (mg/mL).

The study showed that most of the smoothies produced exhibited a greater capacity to inhibit α-amylase compared to the base juice. Only the product with a 6% addition of fresh red currant pomace was less effective. After three months of storage, the α-amylase inhibition capacity decreased in all products except those with a 6% addition of both fresh and dried red currant pomace. This general trend persisted, with apple juice remaining the least effective compared to the others.

The majority of the products examined in this study exhibited a more potent α-amylase inhibitory effect compared to those tested by Nowicka et al. [50]. Cherry juice had an IC_50_ of 2.05 mg/mL, while peach and plum purées showed values of 4.29 and 8.03 mg/mL, respectively. Additionally, most smoothie variants, formed by blending cherry juice with peach or plum purée in ratios of 4:1, 1:1, and 1:4, displayed IC_50_ values ranging between 1.98 and 7.76 mg/mL. Conversely, Nowicka et al. [50] reported IC_50_ values of less than 1.00 mg/mL for apricot purée and smoothies resulting from its combination with cherry juice in ratios of 1:1 and 4:1.

For α-glucosidase inhibition, most smoothies, except those with 6% and 12% fresh red currant pomace and 6% fresh black currant pomace, exhibited greater effectiveness than the base juice. However, in most stored products, excluding those with 3% dried and 6% fresh red currant pomace and 6% dried black currant pomace, a decline in α-glucosidase inhibitory activity was observed. Additionally, after storage, the activities of products with 12% fresh red currant pomace as well as 3% dried and 6% fresh black currant pomace decreased compared to that of apple juice.

The prepared products demonstrated higher effectiveness against α-glucosidase compared to the cherry juice and purées of apricots, plums, and peaches tested by Nowicka et al. [50], whose IC_50_ values were 1.20, 3.47, 4.83, and 6.39 mg/mL, respectively. They also observed less effective inhibition of this enzyme in the case of smoothies made by combining the aforementioned purées with cherry juice in variable proportions (4:1, 1:1, and 1:4), with IC_50_ values ranging between 3.39 and 6.94 mg/mL.

Statistical analysis indicated that the use of red currant pomace resulted in the creation of smoothies with significantly greater effectiveness in inhibiting both enzymes. In terms of α-glucosidase inhibition, the use of dried press waste proved to be significantly more beneficial. Beverages with a single dose of press waste inhibited α-amylase more effectively, while a double dose inhibited α-glucosidase. Storage led to a significant reduction in both activities in the products.

In the case of unstored products, moderate dependencies were noted between the IC_50_ value of the products’ ability to inhibit α-amylase and the content of anthocyanins (*r* = −0.41), flavonols (*r* = −0.44), procyanidin polymers (*r* = −0.49), and total polyphenols (*r* = −0.47) (Supplementary Table S6). However, these dependencies were not observed when analyzing beverages before and after storage (Supplementary Table S5).

### 3.6. Sensory Evaluation

**Figure 1:**
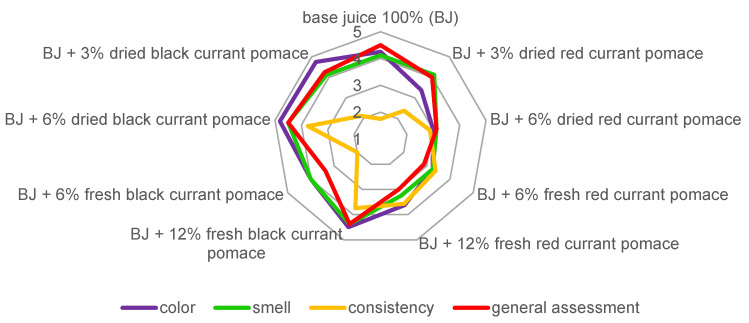
Sensory evaluation of base juice (BJ) and products with the addition of red and black currant pomaces.

**Figure 2:**
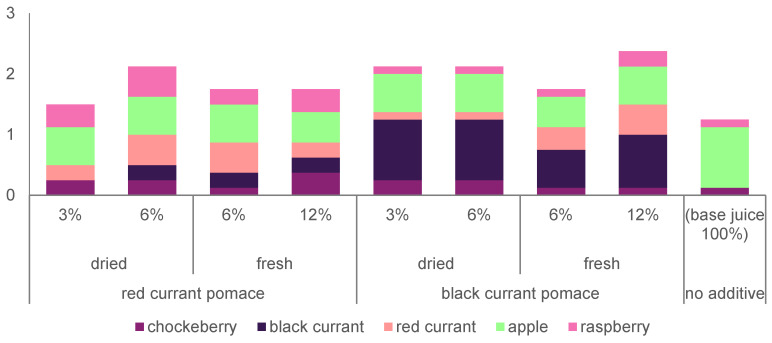
Average amount of fruit flavors determined in the products.

To determine the potential market acceptance of the products, a sensory evaluation was conducted wherein the panelists assessed the color, aroma, overall impression, consistency (Figure 1), and fruit flavors (Figure 2) of the products.

A three-factor statistical analysis showed a significant impact of the type of pomace used on the color assessment of the products. The panelists found the color of smoothies with red currant pomace (rated 3.3) significantly less appealing compared to apple juice (rated 4.2) and products with black currant pomace (rated 4.5), with no statistically significant differences observed between the latter two.

Interestingly, Kucharska et al. [37] found similar results when examining cornelian cherry purées with the addition of chokeberry, raspberry, and strawberry. They noted that the darkest variants (L* values of 31.04 for purées with 10% chokeberry and 28.98 for those with 20% chokeberry) received the highest ratings (4.90 and 5.00, respectively) compared to other variants (L* values ranging from 36.21 to 36.75; color assessments between 4.15 and 4.80) and the basic purée (L* = 36.15; color assessment = 4.80).

Regarding the aroma of the prepared products, a similar pattern emerged as observed in the color assessment. Panelists found that smoothies with red currant pomace had a significantly less appealing aroma (rated 3.4) compared to others (base juice: 4.1; products with black currant pomace: 4.2), with no statistically significant differences noted between the latter two.

Statistical analysis showed that incorporating pomace led to the development of products described as significantly thicker by consumers (red currant pomace: 2.9; black currant pomace: 3.0) compared to the base juice (1.8). However, the differences in the densities between smoothies with both types of pomaces were not statistically significant.

The data collected from assessing the detectability of fruit flavors in the products suggest that their presence was noticeable, but the panelists encountered challenges in unequivocally identifying them. The amount of fruit flavors determined in the products often exceeded the number of raw materials used in their production. For instance, one respondent identified raspberry and chokeberry flavors even in apple juice without any additives. In products containing red currant pomace, the average number of flavors detected ranged from 1.5 to 2.1, while in those with black currant pomace, it ranged from 1.8 to 2.4. On average, more than 2.0 fruit flavors were recognized in 50% of the smoothies tested, with some respondents even detecting 3–5 fruit flavors. The most commonly identified flavor in the variants with red currant pomace and apple juice was apple, while in products with black currant pomace, it was black currant. Nevertheless, using both types of pomaces undoubtedly resulted in products that provided a different sensory experience to consumers compared to the base juice, as respondents perceived more fruit flavors in them (on average between 1.5 and 2.4) than in the juice (on average: 1.2).

**Figure 3:**
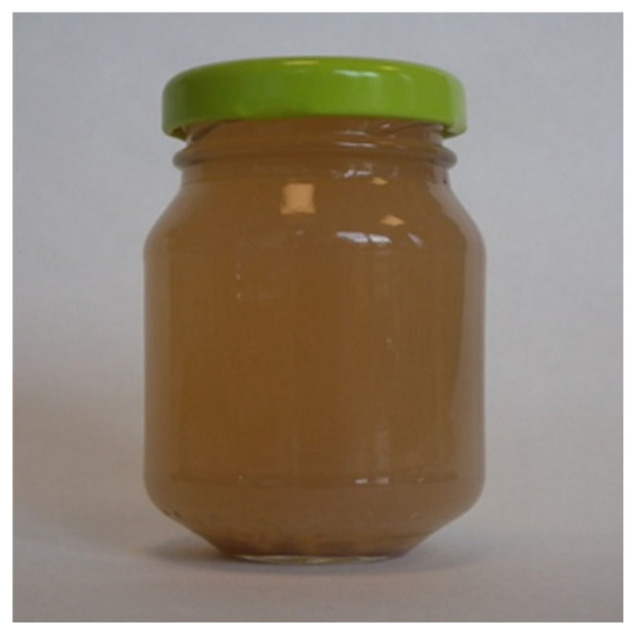
The product with the most favorable overall ratings (4.5): apple juice.

**Figure 4:**
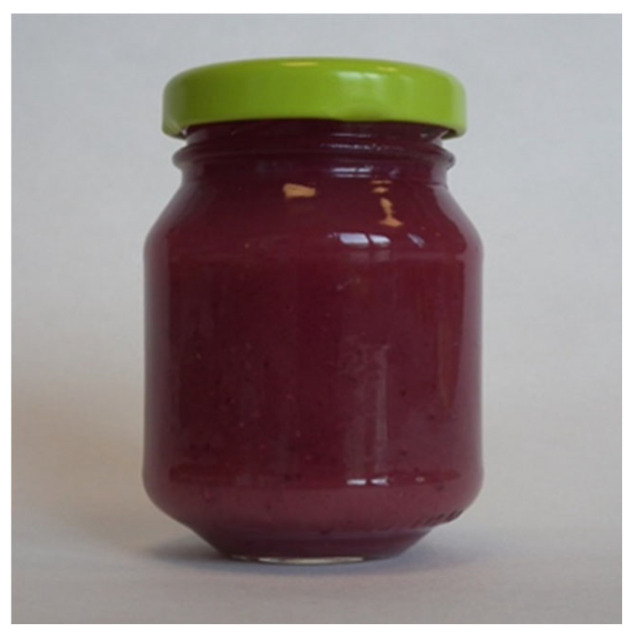
The product with the most favorable overall ratings (4.5): smoothie with 6% dried black currant pomace.

The overall ratings of smoothies fortified with pomace were found to be dependent on the type of pomace used, its processing, and the correlation between the type and dose. The unfortified juice (Figure 3) received the most favorable score (4.5), possibly due to the familiarity of this product type with the average consumer. Interestingly, the beverage containing 6% dried black currant pomace (Figure 4) also received the same score (4.5). Smoothie variants with black currant pomace obtained an average score of 4.1, while those with red currant pomace received a lower score of 3.2. The differences in average ratings were statistically significant. However, it is essential to note that individual ratings for the smoothies ranged from 2.9 to 4.5, indicating that all were above the midpoint of the scale. Moreover, 4 out of 8 products with added pomace received a rating equal to or higher than 4.0 (6% dried red currant pomace, 3% and 6% dried black currant pomace, and 12% fresh black currant pomace). This suggests that they have a high likelihood of finding a broad audience if introduced into the market.

## 4. Conclusions

Enriching apple juice with black and red currant pomaces represents an intriguing avenue for their utilization. This not only offers a solution to repurpose waste generated by the fruit industry during the production of juices, syrups, and wines but also enables the creation of a novel product with elevated levels of bioactive compounds and health-promoting potential.

The examined smoothies containing pomace exhibited modified chemical compositions and physical attributes compared to the original juice. These products showcased a heightened antioxidant capacity and in vitro antidiabetic properties, which hold particular significance for foods that may be targeted toward individuals with diabetes. To validate these findings in real-world settings, conducting in vivo tests would be beneficial to determine whether they result in a smaller increase in the blood glucose levels of people who consume them.

The study showed that the use of black currant pomace allowed for the creation of variants with significantly higher contents of total polyphenols, anthocyanins, flavonols, and procyanidin polymers compared to red currant pomace. These products were also characterized by a significantly higher in vitro antioxidant activity, as confirmed by two of the three methods. In turn, the introduction of red currant pomace resulted in smoothies with significantly higher concentrations of phenolic acids, flavonols, and flavan-3-ols, higher antioxidant properties of FRAP, and more effective in vitro inhibition of α-amylase and α-glucosidase.

The introduction of dried pomace led to the production of smoothies with higher contents of total polyphenols, phenolic acids, flavan-3-ols, and procyanidin polymers compared to the use of fresh additives. Products with dried pomace also showed a significantly higher in vitro antioxidant activity, as confirmed by two methods, as well as more intensive inhibition of α-glucosidase. No significant differences in α-amylase inhibition were noted between the use of dried and fresh pomaces.

It is worth emphasizing that the two products that received the highest overall ratings (4.5) during the sensory evaluation, i.e., the smoothie with 6% dried black currant pomace and apple juice, did not differ significantly in the amounts of total sugars, but the concentration of total organic acids in the enriched product was almost three times higher. This smoothie, together with the product containing 12% fresh black currant pomace, also contained the highest number of total polyphenolic compounds and procyanidin polymers. It also ranked second its anthocyanin, flavonol, and flavan-3-ol contents. Its ABTS antioxidant activity was the most effective. What is more, it was in the second homogeneous group in terms of ORAC antioxidant activity and α-amylase inhibition.

From a technological point of view, the use of dried pomace may potentially be more beneficial than fresh pomace, as it might have a longer shelf life due to its reduced water activity. Therefore, the prospect of producing a variant with 6% dried black currant pomace seems additionally attractive.

However, it should be remembered that all evaluated smoothies offered consumers a distinct sensory experience compared to juice, and they were well received, indicating potential market acceptance. Hence, this study could serve as a foundation for further research investigating the effects of such products on human health and as guidance for food producers.

## Figures and Tables

**Table 1:** Physicochemical parameters of tested products. Letters indicate homogeneous groups determined by a four-factor analysis of variance (ANOVA) using the Duncan test (*p* ≤ 0.05).

Parameter	Storage Time	Apple Juice with								Apple Juice
		Dried Red Currant Pomace		Fresh Red Currant Pomace		Dried Black Currant Pomace		Fresh Black Currant Pomace		
		3%	6%	6%	12%	3%	6%	6%	12%	100%
L* (lightness of color)	0 months	40.64 ± 0.31 a	43.11 ± 0.38 a	50.20 ± 0.11 a	50.86 ± 0.01 a	37.50 ± 0.01 a	35.31 ± 0.01 a	37.13 ± 0.01 a	34.80 ± 0.02 a	37.48 ± 0.05 a
	3 months	39.76 ± 1.63 a	43.64 ± 0.12 a	49.40 ± 0.29 a	51.50 ± 0.17 a	39.22 ± 0.41 a	37.16 ± 0.04 a	38.35 ± 0.08 a	36.30 ± 0.05 a	37.48 ± 0.13 a
a* (intensity of red color)	0 months	13.98 ± 0.15 c	16.60 ± 0.28 a	12.50 ± 0.08 d	15.12 ± 0.01 b	13.84 ± 0.02 c	13.88 ± 0.00 c	15.64 ± 0.01 b	15.27 ± 0.00 b	0.32 ± 0.01 h
	3 months	8.24 ± 0.71 g	14.12 ± 0.05 c	9.88 ± 0.35 f	12.50 ± 0.16 d	10.98 ± 0.49 e	12.28 ± 0.15 d	11.38 ± 0.25 e	13.48 ± 0.19 c	0.12 ± 0.02 h
b* (intensity of yellow color)	0 months	10.39 ± 0.34 a	11.47 ± 0.24 a	9.60 ± 0.03 a	9.85 ± 0.00 a	4.81 ± 0.03 a	3.88 ± 0.01 a	4.22 ± 0.01 a	3.73 ± 0.01 a	3.70 ± 0.04 a
	3 months	7.86 ± 1.99 a	12.25 ± 0.07 a	9.70 ± 0.36 a	10.95 ± 0.20 a	6.36 ± 0.38 a	5.14 ± 0.04 a	5.43 ± 0.20 a	4.14 ± 0.14 a	4.60 ± 0.11 a
Dynamic viscosity (mPas)	0 months	6.0 ± 1.7 a	24.6 ± 0.8 a	137.4 ± 5.9 a	434.3 ± 1.7 a	90.0 ± 1.7 a	350.3 ± 18.7 a	76.2 ± 2.5 a	345.5 ± 8.5 a	5.4 ± 0.8 a
	3 months	6.0 ± 0.0 a	33.0 ± 2.5 a	154.8 ± 1.7 a	451.1 ± 23.8 a	102.0 ± 5.1 a	352.1 ± 9.3 a	85.8 ± 5.9 a	329.3 ± 21.2 a	1.2 ± 0.0 a
Turbidity stability (% NTU)	0 months	10.98 ± 0.23 f	4.35 ± 0.30 i	3.15 ± 0.01 j	4.82 ± 0.37 i	15,98 ± 0.06 c	18.95 ± 0.55 b	18.42 ± 0.11 b	13.27 ± 0.37 e	19.04 ± 0.09 b
	3 months	7.88 ± 0.77 g	6.43 ± 0.01 h	2.30 ± 0.07 jk	1.66 ± 0.18 k	13.68 ± 0.37 e	22.82 ± 0.50 a	13.52 ± 0.78 e	11.72 ± 1.22 f	14.84 ± 0.27 d
Dry matter (g/100 g)	0 months	15.41 ± 0.13 fg	18.72 ± 0.27 a	15.37 ± 0.04 fg	17.01 ± 0.17 d	15.52 ± 0.09 f	17.76 ± 0.03 b	14.22 ± 0.16 i	16.05 ± 0.01 e	13.28 ± 0.11 j
	3 months	15.10 ± 0.03 h	17.91 ± 0.16 b	15.25 ± 0.05 gh	17.11 ± 0.01 d	15.11 ± 0.18 h	17.50 ± 0.11 c	14.36 ± 0.16 i	15.84 ± 0.17 e	13.03 ± 0.04 j
General extract (°Brix)	0 months	13.6 ± 0.0 e	14.9 ± 0.1 a	12.5 ± 0.0 j	12.7 ± 0.0 i	13.0 ± 0.0 g	13.9 ± 0.0 d	12.5 ± 0.0 j	12.1 ± 0.0 l	12.3 ± 0.0 k
	3 months	13.6 ± 0.0 e	14.7 ± 0.0 b	12.5 ± 0.0 j	12.9 ± 0.0 h	13.2 ± 0.0 f	14.3 ± 0.0 c	12.3 ± 0.0 k	11.6 ± 0.1 m	12.5 ± 0.0 j
pH	0 months	3.188 ± 0.004 def	3.206 ± 0.060 def	3.328 ± 0.079 c	3.238 ± 0.023 de	3.141 ± 0.010 f	3.061 ± 0.015 g	3.187 ± 0.030 def	3.134 ± 0.016 f	3.330 ± 0.023 bc
	3 months	3.255 ± 0.017 bd	3.325 ± 0.074 c	3.407 ± 0.013 a	3.427 ± 0.047 a	3.406 ± 0.041 a	3.166 ± 0.016 ef	3.181 ± 0.011 ef	3.130 ± 0.008 f	3.409 ± 0.023 a
Total acidity (g malic acid/100 g)	0 months	0.54 ± 0.03 a	0.70 ± 0.01 a	0.51 ± 0.03 a	0.64 ± 0.03 a	0.56 ± 0.00 a	0.74 ± 0.02 a	0.53 ± 0.00 a	0.69 ± 0.00 a	0.37 ± 0.05 a
	3 months	0.55 ± 0.02 a	0.69 ± 0.00 a	0.50 ± 0.01 a	0.59 ± 0.01 a	0.53 ± 0.00 a	0.69 ± 0.06 a	0.53 ± 0.00 a	0.67 ± 0.06 a	0.35 ± 0.02 a
Ash content (g/100 g)	0 months	0.26 ± 0.04 a	0.29 ± 0.02 a	0.22 ± 0.01 a	0.31 ± 0.01 a	0.24 ± 0.06 a	0.30 ± 0.08 a	0.23 ± 0.01 a	0.30 ± 0.10 a	0.18 ± 0.02 a
	3 months	0.25 ± 0.01 a	0.30 ± 0.01 a	0.23 ± 0.01 a	0.35 ± 0.27 a	0.22 ± 0.03 a	0.27 ± 0.01 a	0.21 ± 0.03 a	0.22 ± 0.08 a	0.17 ± 0.06 a
Pectin content (%)	0 months	0.08 ± 0.01 a	0.10 ± 0.00 a	0.07 ± 0.01 a	0.07 ± 0.01 a	0.07 ± 0.01 a	0.09 ± 0.01 a	0.04 ± 0.03 a	0.07 ± 0.01 a	0.00 ± 0.00 a
	3 months	0.03 ± 0.00 a	0.06 ± 0.01 a	0.03 ± 0.00 a	0.05 ± 0.00 a	0.04 ± 0.01 a	0.08 ± 0.06 a	0.07 ± 0.00 a	0.10 ± 0.04 a	0.02 ± 0.00 a

**Table 2:** The sugar and organic acid contents of products tested. Letters indicate homogeneous groups determined by a three-factor (sugars content) or four-factor (organic acids) analysis of variance (ANOVA) using the Duncan test (*p* ≤ 0.05).

Parameter	Storage Time	Apple Juice with								Apple Juice
		Dried Red Currant Pomace		Fresh Red Currant Pomace		Dried Black Currant Pomace		Fresh Black Currant Pomace		
		3%	6%	6%	12%	3%	6%	6%	12%	100%
Fructose (g/100 g)	0 months	13.28 ± 0.13 b	13.97 ± 0.31 a	12.31 ± 0.12 c	11.35 ± 0.25 f	11.70 ± 0.12 de	11.63 ± 0.26 def	11.41 ± 0.11 ef	10.32 ± 0.23 g	11.85 ± 0.12 d
Sorbitol (g/100 g)	0 months	0.07 ± 0.00 b	0.09 ± 0.01 a	0.09 ± 0.01 a	0.08 ± 0.01 ab	0.08 ± 0.01 ab	0.07 ± 0.00 b	0.09 ± 0.01 a	0.00 ± 0.00 c	0.08 ± 0.01 ab
Glucose (g/100 g)	0 months	2.69 ± 0.06 b	2.97 ± 0.07 a	2.36 ± 0.05 c	2.36 ± 0.05 c	2.24 ± 0.05 de	2.28 ± 0.05 cd	2.17 ± 0.05 ef	2.12 ± 0.05 f	2.18 ± 0.05 ef
Sucrose (g/100 g)	0 months	0.29 ± 0.01 a	0.23 ± 0.01 b	0.00 ± 0.01 e	0.00 ± 0.00 e	0.17 ± 0.00 d	0.20 ± 0.01 c	0.18 ± 0.01 d	0.00 ± 0.01 e	0.22 ± 0.01 bc
Total sugar (g/100 g)	0 months	16.33 ± 0.20 b	17.26 ± 0.39 a	14.76 ± 0.20 c	13.79 ± 0.31 e	14.19 ± 0.18 de	14.18 ± 0.31 de	13.85 ± 0.18 e	12.44 ± 0.28 f	14.33 ± 0.18 cd
Oxalic acid (g/100 g)	0 months	0.00 ± 0.01 c	0.00 ± 0.01 c	0.00 ± 0.01 c	0.00 ± 0.01 c	0.01 ± 0.01 bc	0.02 ± 0.00 b	0.02 ± 0.00 b	0.09 ± 0.00 a	0.00 ± 0.01 c
	3 months	0.00 ± 0.01 c	0.00 ± 0.01 c	0.00 ± 0.01 c	0.00 ± 0.01 c	0.00 ± 0.01 c	0.00 ± 0.01 c	0.00 ± 0.01 c	0.01 ± 0.01 bc	0.00 ± 0.01 c
Maleic acid (g/100 g)	0 months	0.04 ± 0.00 a	0.06 ± 0.02 a	0.04 ± 0.00 a	0.08 ± 0.00 a	0.01 ± 0.01 a	0.02 ± 0.00 a	0.01 ± 0.01 a	0.02 ± 0.00 a	0.00 ± 0.01 a
	3 months	0.00 ± 0.01 a	0.01 ± 0.01 a	0.00 ± 0.01 a	0.00 ± 0.01 a	0.00 ± 0.01 a	0.01 ± 0.01 a	0.01 ± 0.01 a	0.01 ± 0.01 a	0.00 ± 0.01 a
Citric acid (g/100 g)	0 months	0.71 ± 0.02 f	1.32 ± 0.03 b	0.82 ± 0.01 e	2.00 ± 0.02 a	0.62 ± 0.01 h	0.99 ± 0.01 d	0.70 ± 0.02 f	1.28 ± 0.03 c	0.05 ± 0.00 m
	3 months	0.23 ± 0.01 l	0.45 ± 0.01 j	0.24 ± 0.01 l	0.44 ± 0.01 j	0.24 ± 0.01 l	0.67 ± 0.02 g	0.35 ± 0.01 k	0.57 ± 0.02 i	0.02 ± 0.00 n
Malic acid (g/100 g)	0 months	1.42 ± 0.03 f	1.92 ± 0.05 c	1.53 ± 0.04 e	2.51 ± 0.06 a	1.70 ± 0.04 d	1.68 ± 0.02 d	1.68 ± 0.02 d	2.05 ± 0.02 b	0.96 ± 0.02 hi
	3 months	0.95 ± 0.02 i	1.00 ± 0.03 h	0.86 ± 0.01 j	0.86 ± 0.02 j	0.89 ± 0.01 j	1.15 ± 0.03 g	1.13 ± 0.02 g	0.95 ± 0.01 i	0.85 ± 0.02 j
Quinic acid (g/100 g)	0 months	0.00 ± 0.01 d	0.00 ± 0.01 d	0.00 ± 0.01 d	0.00 ± 0.01 d	0.00 ± 0.01 d	0.00 ± 0.01 d	0.00 ± 0.01 d	0.09 ± 0.01 b	0.00 ± 0.01 d
	3 months	0.00 ± 0.01 d	0.00 ± 0.01 d	0.00 ± 0.01 d	0.00 ± 0.01 d	0.00 ± 0.01 d	0.11 ± 0.01 a	0.02 ± 0.01 c	0.00 ± 0.01 d	0.00 ± 0.01 d
Shikimic acid (g/100 g)	0 months	0.02 ± 0.00 a	0.04 ± 0.00 a	0.02 ± 0.00 a	0.06 ± 0.02 a	0.01 ± 0.01 a	0.01 ± 0.00 a	0.01 ± 0.01 a	0.01 ± 0.00 a	0.00 ± 0.01 a
	3 months	0.01 ± 0.01 a	0.02 ± 0.00 a	0.01 ± 0.01 a	0.02 ± 0.01 a	0.00 ± 0.01 a	0.01 ± 0.01 a	0.01 ± 0.01 a	0.00 ± 0.00 a	0.00 ± 0.01 a
Total organic acids (g/100 g)	0 months	2.19 ± 0.07 f	3.34 ± 0.12 c	2.41 ± 0.07 e	4.65 ± 0.12 a	2.35 ± 0.10 e	2.72 ± 0.04 d	2.42 ± 0.06 e	3.54 ± 0.05 b	1.01 ± 0.06 k
	3 months	1.19 ± 0.06 j	1.48 ± 0.06 h	1.11 ± 0.05 jk	1.32 ± 0.07 i	1.13 ± 0.05 jk	1.95 ± 0.09 g	1.52 ± 0.06 h	1.54 ± 0.05 h	0.87 ± 0.06 l

**Table 3:** The polyphenolic compound contents of products tested. Letters indicate homogeneous groups determined by a four-factor analysis of variance (ANOVA) using the Duncan test (*p* ≤ 0.05).

Parameter	Storage Time	Apple Juice with								Apple Juice
		Dried Red Currant Pomace		Fresh Red Currant Pomace		Dried Black Currant Pomace		Fresh Black Currant Pomace		
		3%	6%	6%	12%	3%	6%	6%	12%	100%
Anthocyanins (mg/100 mL)	0 months	1.14 ± 0.03 l	1.98 ± 0.05 j	1.67 ± 0.01 k	2.65 ± 0.08 h	5.26 ± 0.13 d	9.65 ± 0.10 b	6.78 ± 0.07 c	12.80 ± 0.13 a	0.34 ± 0.01 n
	3 months	0.22 ± 0.01 o	0.47 ± 0.01 m	0.37 ± 0.01 n	1.07 ± 0.02 l	2.28 ± 0.06 i	4.55 ± 0.07 f	3.41 ± 0.05 g	4.79 ± 0.12 e	0.04 ± 0.00 p
Phenolic acids (mg/100 mL)	0 months	5.74 ± 0.14 e	8.07 ± 0.20 a	8.16 ± 0.20 a	6.73 ± 0.10 c	6.23 ± 0.09 d	6.17 ± 0.01 d	5.05 ± 0.08 f	5.11 ± 0.08 f	5.18 ± 0.08 f
	3 months	6.09 ± 0.09 d	7.13 ± 0.11 b	4.33 ± 0.06 g	6.83 ± 0.10 c	8.19 ± 0.12 a	2.48 ± 0.01 h	1.23 ± 0.03 j	1.72 ± 0.04 i	6.70 ± 0.17 c
Flavonols (mg/100 mL)	0 months	0.11 ± 0.00 k	0.32 ± 0.01 h	0.09 ± 0.01 k	0.11 ± 0.01 k	0.69 ± 0.02 e	0.98 ± 0.01 c	0.92 ± 0.02 d	1.72 ± 0.03 a	0.00 ± 0.01 m
	3 months	0.14 ± 0.00 j	0.34 ± 0.01 h	0.03 ± 0.00 l	0.20 ± 0.01 i	0.49 ± 0.01 g	1.05 ± 0.03 b	0.52 ± 0.01 f	0.54 ± 0.01 f	0.00 ± 0.01 m
Flavan-3-ols monomeric & dimric (mg/100 mL)	0 months	3.58 ± 0.09 k	10.83 ± 0.16 a	6.20 ± 0.09 d	5.82 ± 0.09 e	3.12 ± 0.08 mn	8.22 ± 0.12 b	3.04 ± 0.01 n	4.02 ± 0.06 j	3.39 ± 0.01 l
	3 months	5.63 ± 0.14 f	7.20 ± 0.11 c	3.27 ± 0.08 lm	5.29 ± 0.08 g	3.94 ± 0.06 j	7.30 ± 0.18 c	4.92 ± 0.07 i	3.35 ± 0.05 l	5.10 ± 0.13 h
Procyanidin polymers (mg/100 mL)	0 months	57.26 ± 0.86 g	73.10 ± 1.83 e	53.33 ± 0.80 h	68.70 ± 1.72 f	106.16 ± 1.59 c	133.70 ± 3.34 b	79.22 ± 1.19 d	135.92 ± 3.40 b	39.24 ± 0.59 j
	3 months	48.71 ± 0.73 i	47.98 ± 1.20 i	37.32 ± 0.56 j	32.13 ± 0.80 k	58.87 ± 0.88 g	161.70 ± 4.04 a	74.23 ± 1.11 e	80.83 ± 2.02 d	32.09 ± 0.48 k
Total polyphenolic compounds (mg/100 mL)	0 months	67.83 ± 1.13 h	94.30 ± 2.25 d	69.45 ± 1.12 h	84.01 ± 1.99 f	121.46 ± 1.91 c	158.72 ± 3.58 b	95.01 ± 1.36 d	159.57 ± 3.69 b	48.15 ± 0.70 j
	3 months	60.79 ± 0.97 i	63.12 ± 1.43 i	45.32 ± 0.72 jk	45.52 ± 1.01 jk	73.77 ± 1.13 g	177.08 ± 4.33 a	84.31 ± 1.28 f	91.23 ± 2.25 e	43.93 ± 0.79 k

**Table 4:** The health-promoting potential (in vitro antioxidant and antidiabetic effects) of the products tested. Letters indicate homogeneous groups determined by a four-factor analysis of variance (ANOVA) using the Duncan test (*p* ≤ 0.05).

Parameter	Storage Time	Apple Juice with								Apple Juice
		Dried Red Currant Pomace		Fresh Red Currant Pomace		Dried Black Currant Pomace		Fresh Black Currant Pomace		
		3%	6%	6%	12%	3%	6%	6%	12%	100%
ABTS (mmol Trolox/100 mL)	0 months	1.163 ± 0.056 b	1.046 ± 0.045 bc	0.705 ± 0.041 fg	0.729 ± 0.042 ef	1.002 ± 0.062 cd	1.662 ± 0.067 a	0.950 ± 0.374 cd	0.868 ± 0.094 de	0.392 ± 0.043 hi
	3 months	0.724 ± 0.051 ef	0.549 ± 0.061 gh	0.339 ± 0.030 i	0.320 ± 0.017 i	0.576 ± 0.017 fg	0.672 ± 0.038 fg	0.424 ± 0.041 hi	0.672 ± 0.045 fg	0.313 ± 0.015 i
FRAP(mmol Trolox/100 mL)	0 months	0.662 ± 0.002 a	0.620 ± 0.013 b	0.343 ± 0.006 g	0.401 ± 0.009 f	0.219 ± 0.002 j	0.499 ± 0.027 d	0.273 ± 0.004 i	0.552 ± 0.009 c	0.229 ± 0.003 j
	3 months	0.449 ± 0.009 e	0.357 ± 0.010 g	0.124 ± 0.008 l	0.182 ± 0.006 k	0.314 ± 0.006 h	0.485 ± 0.004 d	0.228 ± 0.010 j	0.453 ± 0.017 e	0.192 ± 0.003 k
ORAC (mmol Trolox/100 mL)	0 months	3.791 ± 0.587 a	1.359 ± 0.044 cde	1.770 ± 0.341 bc	1.483 ± 0.366 cd	2.368 ± 1.001 b	2.237 ± 0.842 b	2.315 ± 0.110 b	3.719 ± 0.411 a	0.697 ± 0.034 efgh
	3 months	0.483 ± 0.140 gh	0.443 ± 0.070 gh	0.100 ± 0.032 h	0.257 ± 0.214 h	0.483 ± 0.390 gh	0.581 ± 0.429 fgh	0.051 ± 0.177 h	1.234 ± 0.336 cdef	0.961 ± 0.101 defg
Ability to inhibit α-amylase (IC_50_ as mg/mL)	0 months	0.194 ± 0.004 a	0.920 ± 0.018 b	4.441 ± 0.089 e	0.822 ± 0.016 b	0.932 ± 0.019 b	1.125 ± 0.023 b	0.763 ± 0.015 b	0.628 ± 0.013 ab	3.387 ± 0.068 d
	3 months	0.890 ± 0.013 b	0.767 ± 0.015 b	1.691 ± 0.034 c	1.902 ± 0.038 c	1.614 ± 0.032 c	9.435 ± 0.189 f	3.013 ± 0.060 d	1.094 ± 0.031 b	32.147 ± 0.643 g
Ability to inhibit α-glucosidase (IC_50_ as mg/mL)	0 months	0.072 ± 0.002 b	0.026 ± 0.001 a	0.307 ± 0.005 k	0.222 ± 0.005 i	0.163 ± 0.004 e	0.191 ± 0.005 g	0.202 ± 0.004 h	0.123 ± 0.002 c	0.195 ± 0.003 g
	3 months	0.031 ± 0.001 a	0.132 ± 0.003 d	0.206 ± 0.003 h	0.234 ± 0.007 j	0.238 ± 0.004 j	0.189 ± 0.004 g	0.224 ± 0.005 i	0.175 ± 0.005 f	0.207 ± 0.003 h

## Data Availability

The data supporting this study’s findings are available from the corresponding author (Paulina Nowicka, paulina.nowicka@upwr.edu.pl) upon reasonable request. Therefore, correspondence and requests for materials should be addressed to Paulina Nowicka (Department of Fruit, Vegetable and Plant Nutraceutical Technology, Faculty of Biotechnology and Food Science, Wrocław University of Environmental and Life Sciences, 51-630 Wroclaw, Poland).

## Supplementary Materials

The following supporting information can be downloaded at: https://www.mdpi.com/article/10.3390/foods13172715/s1, Table S1: Relationships between the sugar contents and the dynamic vis-cosity presented using the Pearson correlation coefficient (*r*). Table S2: Relationships between the total organic acid content and pH value or total acidity presented using the Pearson correlation co-efficient (*r*). Table S3: Relationships between the anthocyanins content and the pH value or sugar contents presented using the Pearson correlation coefficient (*r*). Table S4: Relationships between the polyphenolic compounds content and the color parameters presented using the Pearson corre-lation coefficient (*r*). Table S5: Relationships between the content of polyphenolic compounds and the health-promoting potential of products presented using the Pearson correlation coefficient (*r*). Table S6: Relationships between the content of polyphenolic compounds and the ability to inhibit α-amylase and α-glucosidase of unstored products presented using the Pearson correlation coeffi-cient (*r*).
