# Supplementary material for: Black and Red Currant Pomaces as Raw Materials to Create Smoothies with In Vitro Health-Promoting Potential
Source: Foods. 2024 Aug 27;13(17):2715. doi: 10.3390/foods13172715 (PMC11395094; doi:10.3390/foods13172715)
Supplement: Supplementary file 1 [file foods-13-02715-s001.zip › foods-3172850-supplementary.pdf]

Table S1. Relationships between the sugar contents and the dynamic viscosity presented using the Pearson correlation coefficient (r).

The Pearson correlation coefficient (r)	Fructose	Sorbitol	Glucose	Sucrose	Total sugar
Dynamic viscosity	-0.64	-0.46	-0.38	-0.68	-0.63

Table S2. Relationships between the total organic acid content and pH value or total acidity presented using the Pearson correlation coefficient (r).

The Pearson correlation coefficient (r)	pH	Total acidity
Oxalic acid	-0.43	0.33
Maleic acid	-0.30	0.38
Citric acid	-0.49	0.63
Malic acid	-0.55	0.45
Quinic acid	-0.34	0.36
Shikimic acid	-0.07	0.39
Total organic acids	-0.53	0.56

Table S3. Relationships between the anthocyanins content and the pH value or sugar contents presented using the Pearson correlation coefficient (r).

The Pearson correlation coefficient (r)	pH	Fructose	Sorbitol	Glucose	Sucrose	Total sugar
Anthocyanins	-0.72	-0.69	-0.72	-0..51	-0.30	-0.66

Table S4. Relationships between the polyphenolic compounds content and the color parameters presented using the Pearson correlation coefficient (r).

The Pearson correlation coefficient (r)	Anthocyanins	Phenolic acids	Flavonols	Flavan-3-ols monomeric & dimric	Procyanidin polymers	Total polyphenolic compounds
L*	-0.53	0.40	-0.59	0.16	-0.53	-0.52
a*	0.48	0.03	0.47	0.26	0.46	0.48
b*	-0.58	0.48	-0.54	0.40	-0.49	-0.47

Table S5. Relationships between the content of polyphenolic compounds and the health-promoting potential of products presented using the Pearson correlation coefficient (r).

The Pearson correlation coefficient (r)	Antioxidant activity			Ability to inhibit (IC_{50} as mg/ml)	
	ABTS	FRAP	ORAC	α-amylase	α-glucosidase
**Anthocyanins**	0.58	0.34	0.60	-0.24	0.00
**Phenolic acids**	0.17	0.06	0.14	0.03	-0.06
**Flavonols**	0.44	0.34	0.46	-0.21	-0.08
**Flavan-3-ols monomeric & dimric**	0.31	0.45	-0.15	0.04	-0.28
**Procyanidin polymers**	0.58	0.47	0.41	-0.16	-0.09
**Total polyphenolic compounds**	0.61	0.49	0.44	-0.17	-0.10

Table S6. Relationships between the content of polyphenolic compounds and the ability to inhibit α-amylase and α-glucosidase of unstored products presented using the Pearson correlation coefficient (r).

The Pearson correlation coefficient (r)	Unstored products abilities to inhibit (IC_{50} as mg/ml)	
	α-amylase	α-glucosidase
**Anthocyanins**	-0.41	-0.04
**Phenolic acids**	0.35	0.08
**Flavonols**	-0.44	-0.15
**Flavan-3-ols monomeric & dimric**	0.02	-0.25
**Procyanidin polymers**	-0.49	-0.16
**Total polyphenolic compounds**	-0.47	-0.16
